# KneeXNet-2.5D: a clinically-oriented and explainable deep learning framework for MRI-based knee cartilage and meniscus segmentation

**DOI:** 10.1038/s44401-026-00072-5

**Published:** 2026-02-16

**Authors:** Maimouna Sanogo, Fengyi Gao, Nickolas Littlefield, Ismaeel A. Siddiqui, Luke A. Carlson, Amin Rezaei, Pavan Bodanki, Zoe Menezes, Abha Kanda, Mehrnaz Abedian, George M. Mastorakos, Michael R. Kann, Soheyla Amirian, Maedeh Agharazidermani, Arezoo Sarkheyli-Hägele, Kasey Harshman, Nicole Myers, Hilal Maradit Kremers, James J. Irrgang, Brian J. McGrory, Adolph J. Yates, Johannes F. Plate, Ahmad P. Tafti

**Affiliations:** 1 School of Health and Rehabilitation Sciences, University of Pittsburgh, Pittsburgh, PA USA; 2 Intelligent Systems Program, University of Pittsburgh, Pittsburgh, PA USA; 3 Computational Pathology and AI Center of Excellence (CPACE), University of Pittsburgh, Pittsburgh, PA USA; 4 School of Medicine, University of Pittsburgh, Pittsburgh, PA USA; 5 School of Computing, University of Georgia, Athens, GA USA; 6 Scienza Health, Scottsdale, AZ USA; 7 Seidenberg School of Computer Science and Information Systems, Pace University, New York, NY USA; 8 School of Information, Florida State University, Tallahassee, FL USA; 9 Department of Computer Science and Media Technology, Sustainable Digitalization Research Center (SDRC), Malmö University, Malmö, Sweden; 10 Department of Orthopedic Surgery, Mayo Clinic, Rochester, MN USA; 11 Department of Orthopaedic Surgery, Tufts University, Medford, MA USA; 12 Department of Orthopaedic Surgery, University of Pittsburgh, Pittsburgh, PA USA

**Keywords:** Computational biology and bioinformatics, Engineering, Health care, Mathematics and computing, Medical research

## Abstract

Accurate segmentation of knee cartilage and meniscus in magnetic resonance imaging (MRI) is essential for the early detection and monitoring of complications such as cartilage erosion and osteoarthritis. Yet, manual annotation remains time-consuming, subjective, and inefficient for routine clinical use. In this study, we introduced *KneeXNet-2.5D*, a clinically oriented and explainable deep learning framework for accurate and efficient knee cartilage and meniscus segmentation in sagittal MRIs. Unlike traditional 3D segmentation methods, the proposed model employs a 2.5D architecture to capture inter-slice spatial context, achieving high segmentation accuracy while maintaining computational efficiency and optimal resource utilization. We further incorporated targeted image augmentation, including synthetic noise injection, to enhance the AI model robustness against medical imaging variability. The efficient design of the 2.5D model allows for reduced resource consumption, making it suitable for deployment in healthcare settings with limited computational infrastructure, particularly in low-resource hospitals and rural care environments. To enable open scientific research and ensure reproducibility, we constructed a gold-standard, manually segmented knee MRI dataset and publicly released it alongside the annotation guideline, source code, trained AI models, and a lightweight software application. An entropy-based AI explainability strategy was developed to highlight high-uncertainty regions that are most influential to model predictions, advancing transparency and interpretability. Clinical relevance and anatomical validity were further assessed through expert review by board-certified orthopedic surgeons. Together, these contributions demonstrate the AI model’s anatomical fidelity, interpretability, and readiness for integration into musculoskeletal imaging workflows.

## Introduction

Degeneration of knee cartilage and meniscus is a prominent indicator of osteoarthritis (OA), a chronic and degenerative joint disease affecting millions of individuals and posing a significant burden on healthcare systems worldwide^1–6^. Accurate evaluation of knee cartilage integrity is very important for early diagnosis, timely intervention, and longitudinal monitoring of OA and related musculoskeletal complications. Magnetic resonance imaging (MRI) has emerged as the gold standard for non-invasive assessment of knee cartilage and meniscus due to its high soft-tissue contrast and spatial resolution. However, manual segmentation of cartilage and meniscus in knee MRIs remains labor-intensive, subjective, and impractical for large-scale or routine clinical workflows^7–11^. Manual segmentation is also prone to inter- and intra-observer variability, potentially compromising the consistency of diagnostic outcomes and assessments. Moreover, as musculoskeletal imaging volumes continue to grow for clinical follow-up(s), especially in aging populations, reliance on manual interpretation poses scalability challenges that may strain healthcare resources. Automated segmentation methods not only offer the potential to reduce clinical workload but also provide standardized and reproducible measurements that are essential for objective evaluation, treatment planning, and clinical trials. Integrating such tools into clinical practice and research could enable earlier detection of degenerative changes, facilitate more precise surgical planning, and support evidence-based decision-making at scale.

Artificial intelligence (AI) strategies, particularly convolutional neural networks (CNNs), have shown successful applications in automating knee cartilage and meniscus segmentation from MRIs^7,10–18^. These AI-powered methods can be broadly categorized into 2D, 3D, and 2.5D architectures, each offering trade-offs between segmentation accuracy and computational complexity. 3D CNNs provide strong performance by leveraging volumetric spatial context, which is particularly beneficial for anatomically contiguous structures like cartilage. Ambellan et al.^16^ combined 3D statistical shape models with 2D and 3D CNNs for accurate segmentation of knee anatomy. Çiçek et al.^19^ extended the original U-Net^20^ into a 3D segmentation strategy to support dense volumetric segmentation from sparsely annotated images. While such AI-powered models demonstrate strong performance, they often require extensive 3D annotations and high performance computational resources, and may lack robustness across datasets with imaging noise, protocol variability, or anatomical differences. By contrast, 2D CNNs are more computationally efficient and easier to deploy. However, 2D CNNs generally fail to capture cross-slice contextual information, often leading to fragmented or anatomically inconsistent segmentation when applied to 3D volumetric images.

To bridge this gap, 2.5D models have been proposed. These approaches typically use a stack of adjacent 2D slices, such as slices *z* − 1, *z*, and *z* + 1, with z representing slice position, as input to the CNN, thereby capturing limited volumetric context while preserving the computational efficiency of 2D models. Avesta et al.^21^ found that while 3D models performed best overall, 2.5D methods offered a strong trade-off between accuracy and computational feasibility. More recently, Kumar et al.^22^ and Hung et al.^23^ introduced attention-based 2.5D architectures to further improve inter-slice context modeling and segmentation quality across diverse tasks. Despite these advancements, most existing methods address segmentation accuracy, computational efficiency, AI explainability, and generalizability in isolation. Few frameworks have holistically tackled all four dimensions, especially for knee cartilage and meniscus segmentation, with explicit attention to real-world imaging variability, clinical integration, and reproducibility. In addition, the limited availability of high-quality and publicly annotated datasets for knee MRI continues to limit benchmarking, validation, and broader adoption of AI-driven solutions. In light of these challenges, we emphasize the *clinical* and *technical* significance of our contribution, namely *KneeXNet-2.5D*, as follows:

- **Clinical Significance**: *KneeXNet-2.5D* addresses a pressing clinical need for scalable, accurate, and explainable cartilage and meniscus segmentation in routine knee MRI analysis. By reducing the time, resources, and expertise required for manual annotation and enabling standardized quantitative evaluation, our framework has the potential to enhance early knee OA diagnosis, support radiologist workflows, and promote reproducible musculoskeletal imaging research across institutions.
- **Technical Significance**: *KneeXNet-2.5D* leverages a hybrid 2.5D U-Net architecture for efficient and anatomically precise segmentation of knee cartilage and meniscus in MRI. The model is engineered to balance computational efficiency with spatial contextual awareness, while a novel *scale-space representation* framework is incorporated to improve the AI model generalization and robustness. In the *noise-space* dimension, we apply structured Gaussian perturbations, mimicking sensor noise, motion blur, and illumination artifacts, to enforce noise-invariant feature learning. In the *scale-space* dimension, we introduce dynamic resizing to simulate clinical scenarios where anatomical structures vary in size across slices or patient populations. This continuous augmentation strategy ensures the model learns scale-consistent features essential for robust deployment. The pipeline further includes entropy-based AI explainability to identify prediction uncertainty, paired with domain-expert-in-the-loop evaluation for clinical interpretability. Finally, we contribute a gold-standard manually segmented MRI dataset and release the full open-source materials, pretrained AI models, and a lightweight software application to facilitate reproducibility and translational adoption.

**Fig. 1:**
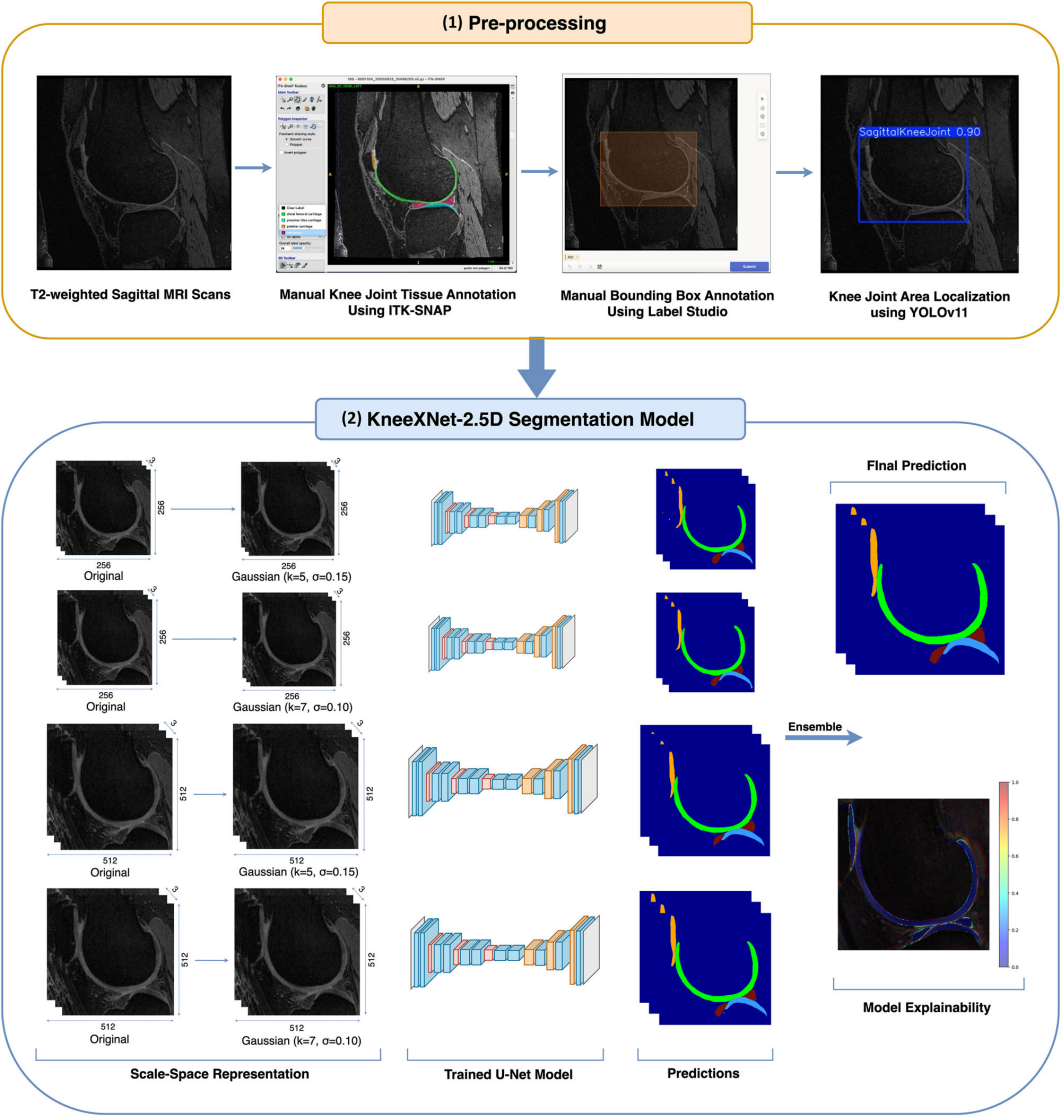
Overview of the proposed framework, the *KneeXNet-2.5D* model, which consists of two main stages. **1** Pre-processing and **2** AI-powered KneeXNet-2.5D segmentation model. The pre-processing stage follows a sequential workflow: starting with T2-weighted sagittal MRI acquisition, followed by manual knee tissue annotation using ITK-SNAP, bounding box annotation using Label Studio, and knee joint area localization guided by YOLOv11. In the segmentation stage, four 2.5D U-Net models are independently trained using distinct data augmentation settings, including a novel *scale-space representation* strategy. This augmentation approach introduces controlled Gaussian noise and systematic scale variation to simulate real-world imaging artifacts and anatomical variability, thereby improving AI model robustness. The softmax outputs from each model are spatially aligned and combined via an ensemble strategy to generate the final segmentation. We further incorporate entropy-based maps to provide AI explainability, visually highlighting regions of uncertainty that influence the AI model’s predictions.

Figure 1 illustrates the overall *KneeXNet-2.5D* pipeline, providing a high-level view of our framework from data pre-processing to AI model development, AI explainability, and clinical validation. This schematic serves as a conceptual roadmap for the study, highlighting the interconnected components built for the proposed system. The detailed methodology, including architectural design, image augmentation strategies, segmentation evaluation, and expert-in-the-loop review, is presented in the subsequent sections.

## Results

In this section, we present a comprehensive evaluation of the proposed *KneeXNet-2.5D* framework across several core components. These include knee joint area localization accuracy, segmentation performance, AI training and validation, robustness under diverse augmentation settings, and AI explainability through entropy-based uncertainty analysis. We also benchmark the computational efficiency of our 2.5D model against conventional 3D segmentation approaches and compare its performance with other state-of-the-art methods. Moreover, we summarize expert validation feedback and showcase qualitative results from our lightweight software application to illustrate its potential for integration into clinical musculoskeletal imaging workflows. Regarding computational resources, our computing node was configured with three NVIDIA Quadro RTX 8000 GPUs, each providing 48 GB of memory and 4,608 CUDA cores. The system also included two CPUs, each with 20 cores, and a total of 376 GB of system memory, enabling efficient support for GPU-accelerated computation. All experiments and AI model development were conducted using the Python (3.10.12) programming language. The framework integrates popular Python-based libraries such as PyTorch (2.6.0) for deep learning model implementation, NumPy (1.26.4) for numerical operations, and Streamlit for building the interactive user interface. This choice ensures high reproducibility, modularity, and ease of deployment across diverse computing environments.

### Knee joint area localization performance

The YOLOv11^24,25^ localization model was trained on a manually annotated subset of sagittal knee MRI slices and initialized using pretrained weights (yolo11n.pt). The trained detector was then applied across the dataset to automatically generate bounding boxes for all slices (see Fig. 1; Pre-processing). The model achieved a mean Average Precision (mAP) of 0.9949, indicating highly accurate localization and classification of regions of interest (ROI) across sagittal MRI slices. This near-perfect score reflects the model’s strong performance in identifying relevant anatomical structures with high spatial precision and minimal false detections.

### Knee cartilage and meniscus segmentation performance

The performance of the AI-powered segmentation was evaluated on the *test set*, as detailed in the Model Training and Image Augmentation section, using Intersection over Union (IoU)^26^ and Dice Similarity Coefficient (DSC)^27^. Evaluation focused on four key anatomical structures: (1) distal femoral cartilage, (2) proximal tibial cartilage, (3) patellar cartilage, and (4) meniscus. The IoU and DSC scores were calculated separately for each structure. To obtain the final performance metrics, the scores across the four segmentation targets were averaged. For each patient, the slice range used in the analysis varied depending on the visibility of the relevant joint structures in the MRI scan. Segmentation began and ended at the slices where these structures were clearly visible, and only those slices were included in the study. This method ensures that performance is assessed across anatomically relevant slices rather than being skewed by large regions containing only background. For evaluation, metrics were computed only on slices that contained cartilage or meniscus, since background-only slices would inflate accuracy and do not reflect anatomical segmentation quality. This slice selection was used solely for metric calculation; the *KneeXNet-2.5D* pipeline processes the entire MRI stack automatically during inference and does not require any manual identification of relevant slices in clinical deployment. The IoU and DSC scores correspond to the segmentation results within this specific slice range for each MRI scan. This method provides a comprehensive evaluation by ensuring that performance is assessed across all relevant structures, rather than being biased toward any single region. Both the *KneeXNet-2.5D-Baseline* and the *KneeXNet-2.5D* consistently achieved promising results across all structures.

**Fig. 2:**
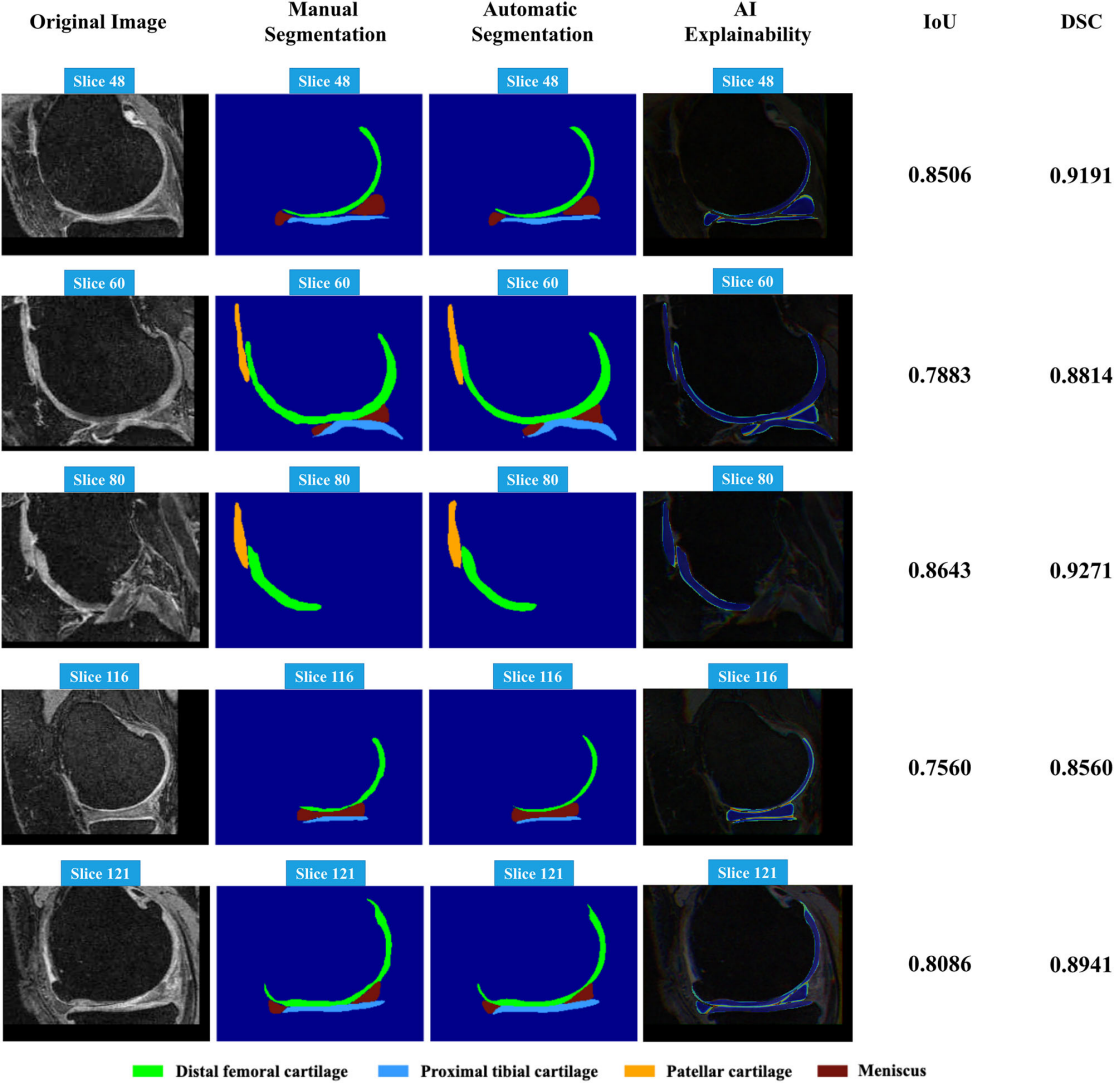
Qualitative Segmentation Results on Sagittal Knee MRI. Predicted segmentation results from the *KneeXNet-2.5D-Baseline* model overlaid on sagittal MRI slices, demonstrating the AI model’s ability to accurately identify cartilage and meniscus structures in representative test cases.

**KneeXNet-2.5D-Baseline:** The baseline model, *KneeXNet-2.5D-Baseline*, was trained on clean images without augmentation. It achieved high segmentation accuracy, with an average IoU of 0.8021 and an average DSC score of 0.8721, as shown in Table 1. These values demonstrate promising spatial agreement between the predicted segmentation masks by the model and the ground-truth annotations, indicating that the model accurately captures anatomical boundaries under standard imaging conditions, as illustrated in Fig. 2.

**Table 1:** AI-powered segmentation and robustness performance of *KneeXNet-2.5D*, *KneeXNet-2.5D-Baseline*, and the individual per-configuration models

Models	IoU	DSC	RI	CRRS
KneeXNet-2.5D-Baseline	0.8021	0.8721	–	–
KneeXNet-2.5D (256, *k* = 5, *σ* = 0.15)	0.7983	0.8700	0.9964	–
KneeXNet-2.5D (256, *k* = 7, *σ* = 0.10)	0.7975	0.8695	0.9956	–
KneeXNet-2.5D (512, *k* = 5, *σ* = 0.15)	0.7879	0.8594	0.9839	–
KneeXNet-2.5D (512, *k* = 7, *σ* = 0.10)	0.7894	0.8616	0.9861	–
KneeXNet-2.5D	0.8108	0.8779	–	0.9992

Each configuration of *KneeXNet-2.5D* was trained with a specific Gaussian blur setting, where 256 or 512 refers to the input image size (in pixels), *k* is the Gaussian kernel size, and *σ* is the standard deviation used in the Gaussian blur. Additional details on the definitions and computation of IoU, DSC, RI, and CRRS are provided in the *Methods* section.

**Fig. 3:**
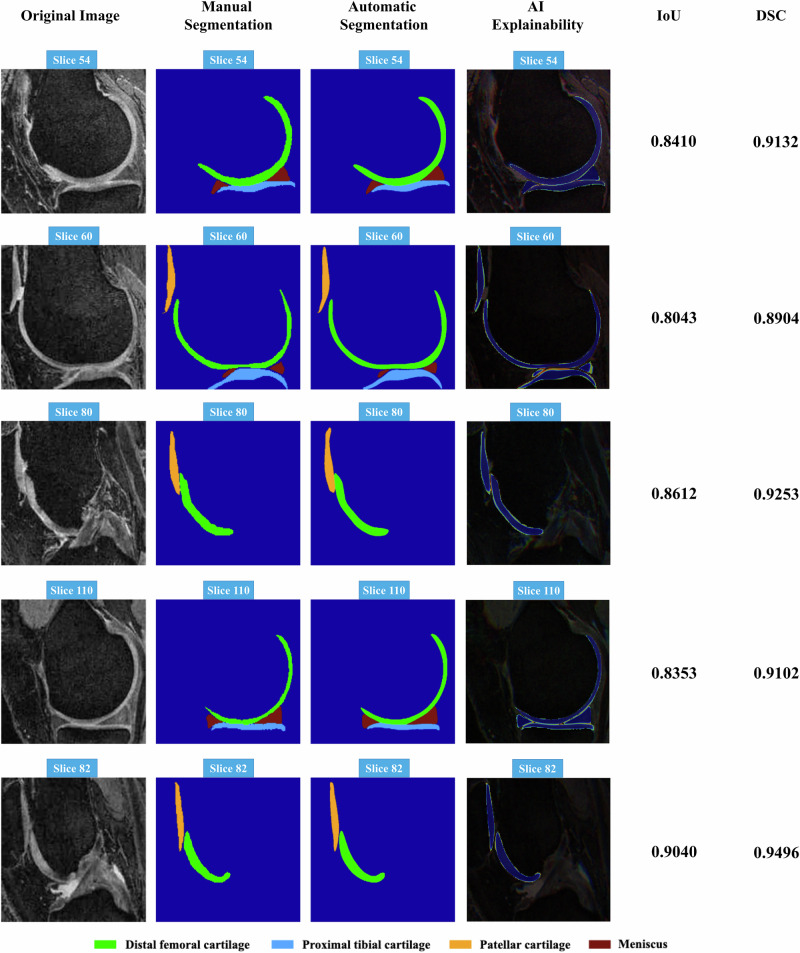
Qualitative Segmentation Results on Sagittal Knee MRI. Predicted segmentation results from the *KneeXNet-2.5D* model overlaid on sagittal MRI slices, showcasing the AI model’s ability to accurately delineate cartilage and meniscus structures in exemplary test cases.

**KneeXNet-2.5D:** The augmented model, *KneeXNet-2.5D*, was trained with additional Gaussian blur augmentations to improve robustness. It achieved comparable segmentation performance to the baseline on clean test data, with an IoU of 0.8108 and a DSC score of 0.8779, as shown in Table 1, indicating that the augmentation strategy achieved high accuracy. The individual models trained with different augmentation settings yielded IoU scores between 0.7879 and 0.7983, and DSC scores between 0.8594 and 0.8700. These demonstrate stable segmentation performance across different image resolutions and blur parameters. *KneeXNet-2.5D* outperformed *KneeXNet-2.5D-Baseline*, with the Wilcoxon signed-rank test^28^ at *α* = 0.05 showing *p* < 0.001 for both paired IoU and Dice scores, indicating a statistically significant difference in segmentation performance. Qualitative segmentation examples are shown in Fig. 3, further supporting the accuracy and robustness of *KneeXNet-2.5D*.

### AI training and validation performance

**Fig. 4:**
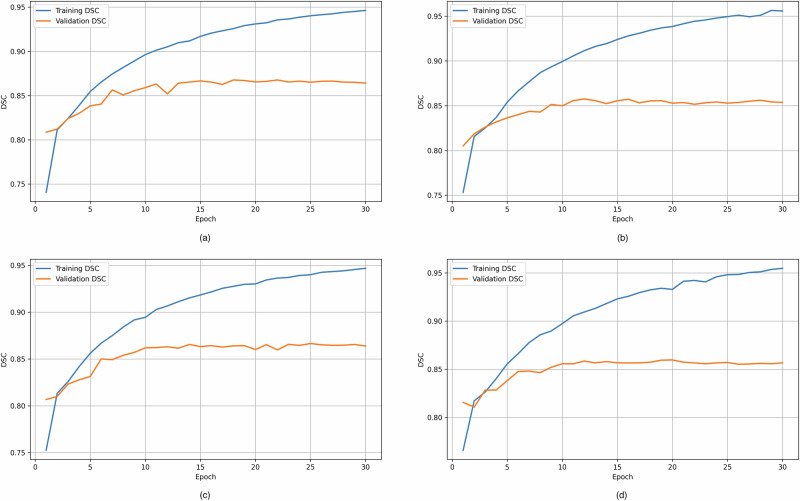
Training and validation DSC curves for *KneeXNet-2.5D* under varying input resolutions (256 × 512) and Gaussian noise levels (*σ* = 0.10, 0.15). Training DSC steadily increases across epochs, while validation DSC plateaus after 10 epochs. **a** Input = 256, *k* = 5, *σ* = 0.15; **b** Input = 512, *k* = 5, *σ* = 0.15; **c** Input = 256, *k* = 7, *σ* = 0.10; **d** Input = 512, *k* = 7, *σ* = 0.10.

**Fig. 5:**
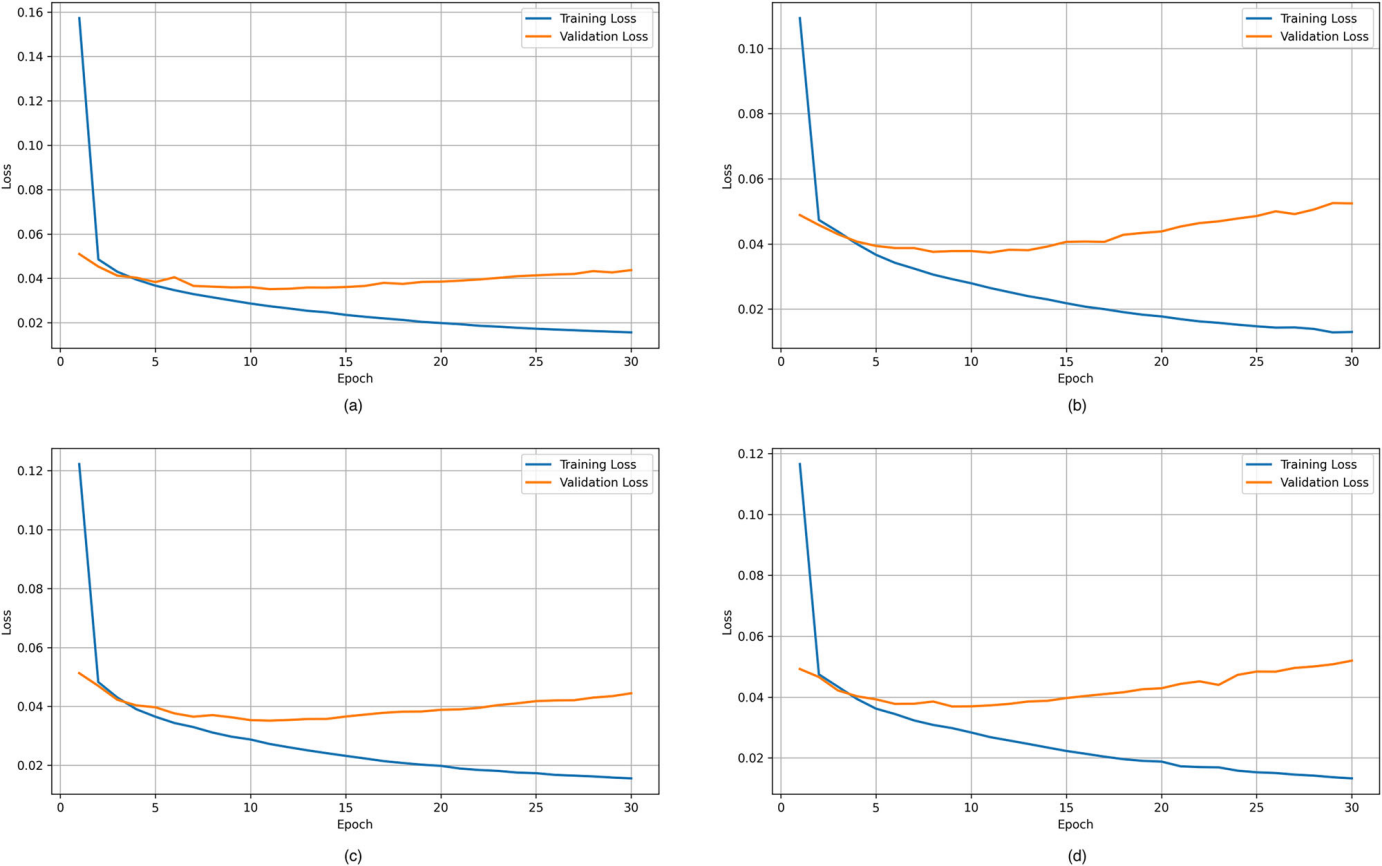
Training and validation loss curves for *KneeXNet-2.5D* under varying input resolutions (256 × 512) and Gaussian noise levels (*σ* = 0.10, 0.15). Across all configurations, training loss continues to decrease while validation loss stabilizes after 15 epochs, indicating the onset of overfitting. **a** Input = 256, *k* = 5, *σ* = 0.15; **b** Input = 512, *k* = 5, *σ* = 0.15; **c** Input = 256, *k* = 7, *σ* = 0.10; **d** Input = 512, *k* = 7, *σ* = 0.10.

During AI model development, we monitored the DSC and loss on both the training and validation sets to assess learning progress. Figure 4 presents the training and validation DSC curves for the four *KneeXNet-2.5D* configurations. Across all settings, the training DSC increased steadily over 30 epochs, reaching around 0.95. All four configurations converged by around 10 epochs. While signs of overfitting appeared after around 15 epochs, the models maintained consistent performance levels. Overall, all four configurations demonstrate fast convergence by 10 epochs and stable model performance with only mild overfitting beyond 15 epochs. Fig. 5 shows the training and validation loss curves for the four *KneeXNet-2.5D* configurations. Based on the training and validation loss curves, all models converge by about 10 epochs. After 15 epochs, the validation loss begins to increase, indicating that the models begin to overfit. Due to the fact that the selected model was based on the highest validation IoU, the chosen model was not overfit despite this trend.

### AI model robustness performance

Model robustness scores, including Robustness Index (RI)^29–31^ and Composite Robustness-Recovery Score (CRRS)^32^, for individual models trained with different image augmentation settings are summarized in Table 1, with values ranging from 0.9839 to 0.9992. These results indicate that all *KneeXNet-2.5D*-related models exhibited strong robustness. Models trained with 256 × 256 input resolution generally outperformed their 512 × 512 counterparts in robustness, suggesting that finer-scale augmentations at lower resolutions may contribute to better generalization. Among all settings, the model trained with a 256 × 256 resolution and kernel size 5 (*σ* = 0.15) achieved the highest robustness score (0.9964), where *σ* is the standard deviation utilized as a key parameter in Gaussian blur, and it was directly tied to how much blurring occurred. The augmented model, *KneeXNet-2.5D*, obtained a CRRS of 0.9992, as shown in Table 1. The result indicates that the *KneeXNet-2.5D* model provides reliable and robust segmentation performance by leveraging the strengths of individual augmentation configurations. This result also shows that the augmentation strategy, particularly when applied with appropriate resolution and blur parameters, enhances model robustness without compromising baseline accuracy. Further details on the definitions and computation of RI and CRRS can be found in the *Methods* section.

### AI explainability performance

**Fig. 6:**
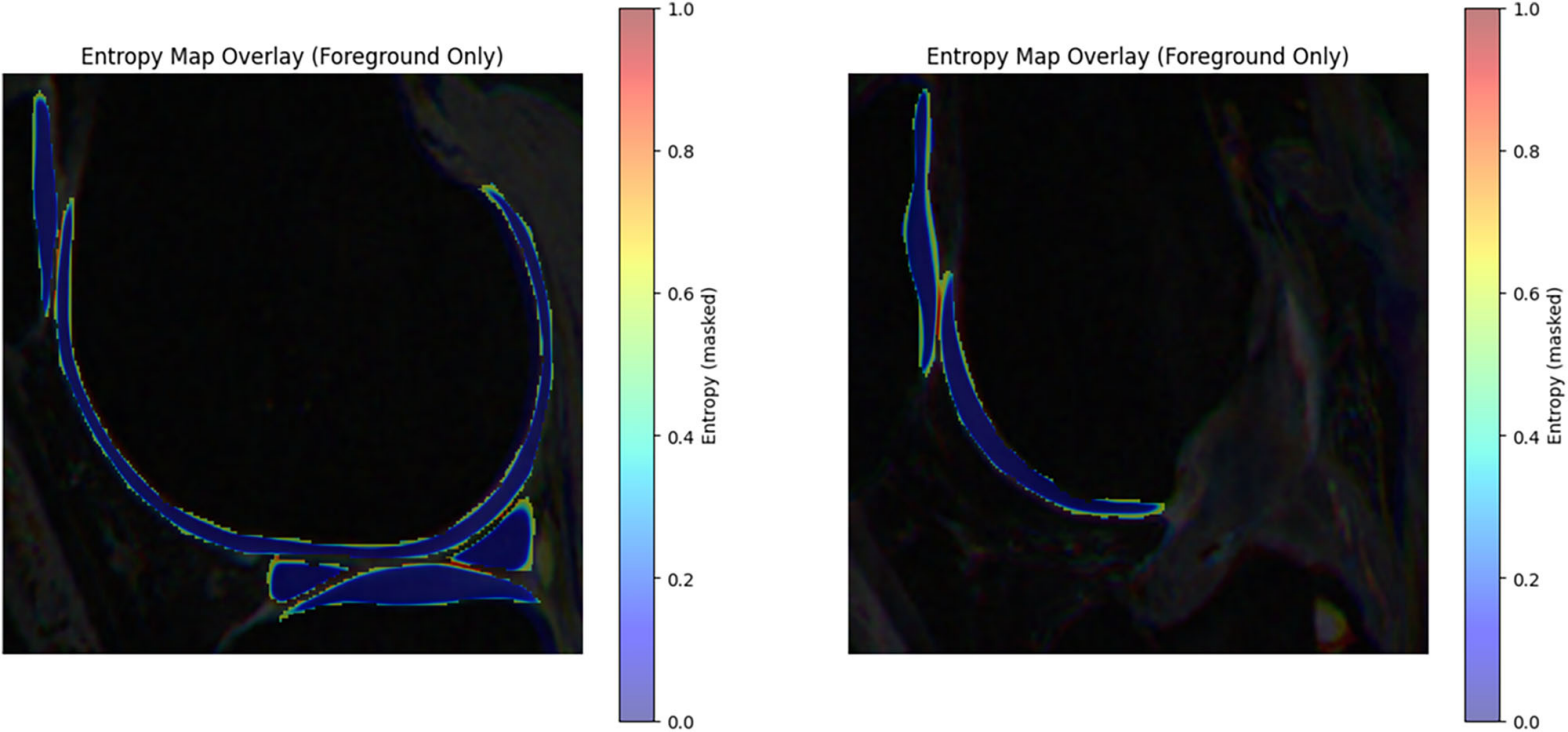
Entropy-based uncertainty maps for AI explainability (two representative examples). The map is computed from the fused softmax outputs of four independently trained models and overlaid on the input MRI slice. Warmer colors indicate high uncertainty, while cooler colors represent confident predictions. Background pixels are masked to focus on uncertainty in foreground anatomical structures.

Figure 6 illustrates the entropy maps^33^ and how confident the model is in different parts of its segmentation. In medical image segmentation, entropy maps will highlight uncertainty in a visual way^34,35^. In entropy maps, low entropy (shown in cooler colors like blue) means the model is confident in its predictions, while high entropy (shown in warmer colors like red) indicates uncertainty is often mapped near anatomical boundaries or in areas with unclear features. In Fig. 6, we see low-entropy regions across most of the cartilage and meniscus, suggesting strong model confidence for the segmentation task. High-entropy areas appear mainly around structure edges, where predictions are more difficult. Even background regions that the model classifies with high certainty show low entropy, confirming that entropy reflects prediction confidence regardless of class. When we mask out background pixels, the uncertain areas within the anatomical structures become easier to see and interpret. This makes the visualization a useful tool for expert-in-the-loop evaluation, offering both intuitive and quantitative insight into the model’s behavior. We evaluated the performance of the proposed AI explainability with two different strategies as follows:

**Faithfulness:** The *KneeXNet-2.5D* model achieved a mean IoU of 0.8108 and a mean DSC of 0.8779. By selectively adding noise to the high-uncertainty (high-entropy) regions, the scores dropped sharply to 0.2521 (IoU) and 0.2560 (DSC), as shown in Table 2. These large performance drops, 0.5586 for IoU and 0.6219 for DSC, highlight the AI model’s sensitivity to targeted corruption and support the faithfulness of the AI explainability method, confirming that the model relies on spatial regions it implicitly identifies as important for the segmentation task.

**Table 2:** Faithfulness evaluation for AI explainability using entropy-based corruption on the fused segmentation output

Metric	Original Input	Corrupted Input	Performance Drop (Δ)
Mean IoU	0.8108	0.2521	−**0.5586**
Mean DSC	0.8779	0.2560	−**0.6219**

Performance drop was computed by corrupting the top 20% high-entropy regions and measuring the decrease in segmentation quality.

**Domain-Experts-in-the-Loop:** Two board-certified orthopedic surgeons with expertise in knee disorders reviewed the AI explainability results of our model. As part of the evaluation, we provided each expert with a random subset of 15 test MRI scans alongside the corresponding segmentation outputs and entropy maps overlaid on corresponding sagittal MRI slices. Their assessment focused on two key aspects: (1) whether the predicted segmentation of cartilage and meniscus were anatomically accurate, and (2) whether the regions of high entropy aligned with clinically relevant or ambiguous structures, such as cartilage edges and meniscal horns. Both experts confirmed that the model’s segmentation was consistent with clinical anatomy and that areas of high uncertainty often corresponded to zones where clinical interpretations may vary. This feedback reinforces the model’s clinical relevance and interpretability in real-world applications.

### AI model efficiency comparison: 3D vs. 2.5D segmentation

The *3D U-Net* model was trained with a batch size of 4, which is the only setting that differs from *KneeXNet-2.5D*, using full volumes standardized to 112 slices of size 224 × 224 after removing 28 lateral slices and 20 medial slices from each sagittal MRI scan to exclude regions without key knee tissues. The experiment used 222 MRI scans, and the dataset was split at the patient level using an 80/10/10 ratio. Specifically, 80% of subjects were assigned to the training set, and the remaining 20% were evenly divided into validation and test sets using a fixed random seed, ensuring that no subject appears in more than one subset. The model was trained for 1000 epochs to compensate for the limited dataset size. Table 3 presents a comparison of the *3D U-Net*, *KneeXNet-2.5D*, and *KneeXNet-2.5D-baseline* models in terms of segmentation accuracy, training time, and inference time. *KneeXNet-2.5D* achieved the highest segmentation performance, with an IoU of 0.8108 and a DSC of 0.8779, outperforming both its *KneeXNet-2.5D-baseline* (IoU: 0.8021, DSC: 0.8721) and the *3D U-Net* (IoU: 0.5428, DSC: 0.5706). The increased training time (569.30 min) and memory usage (10.42 GB) of *KneeXNet-2.5D* relative to its baseline is attributed to its ensemble design. This incorporates multiple models trained under distinct Gaussian blur configurations and varying input resolutions to improve robustness. Compared to the *3D U-Net*, the *KneeXNet-2.5D-baseline* was significantly more efficient, requiring only 81.32lmin of training time and 1.34 GB of memory, whereas the *3D U-Net* required 1357.49 min and 6.53 GB. During inference, the *KneeXNet-2.5D-Baseline* model demonstrated the fastest runtime at 0.42 s and required only 0.35 GB of memory, making it highly efficient for deployment in resource-constrained environments such as clinical edge devices or real-time systems. In contrast, the full *KneeXNet-2.5D* model required significantly more time (8.91 s) and memory (2.38 GB) due to its larger architecture and multi-slice processing, which introduces additional computational overhead. Despite this, it offered higher segmentation accuracy, making it suitable for scenarios where accuracy is prioritized over speed. The *3D U-Net* consumed more inference time (0.50 s) and memory (0.64 GB) than the baseline and produced substantially lower segmentation accuracy. The poor segmentation performance of *3D U-Net* is likely due to the limited size of the gold-standard MRI dataset, which restricts the model’s ability to effectively learn volumetric features, particularly for complex anatomical structures. It is important to note that the 3D U-Net results in Table 3 are provided only as a computational reference rather than a direct competitive baseline. Because the 3D model does not achieve clinically viable segmentation performance under the limited-data setting of our study, its training and inference efficiency should not be interpreted as a fully optimized alternative, but rather as an illustration of the inherent computational cost associated with full 3D processing. Full 3D networks are known to be data-hungry and sensitive to dataset sparsity; thus, without extensive labeled volumes, they tend to overfit or generalize poorly. As discussed earlier, the study included only 222 MRI scans, which were split at the patient level into 80/10/10 training, validation, and test sets using a fixed random seed to prevent subject overlap. To allow fair comparison across methods, we adopted a deliberately simple training configuration; however, this setup does not compensate for the intrinsic data requirements of full 3D architectures. As a result, the *3D U-Net* underperforms from substantially larger datasets. This further highlights the advantages of our proposed *KneeXNet-2.5D* framework, which is designed to maintain strong performance in low-data regimes by leveraging slice-level supervision and more robust training dynamics compared with full 3D architectures. Additionally, the higher computational burden and memory consumption make such models less practical for clinical deployment without access to high-end GPU infrastructure. These results highlight the effectiveness of the proposed 2.5D architecture in achieving a strong trade-off between segmentation performance and computational cost. This positions it as a practical and scalable alternative to full 3D models for volumetric knee segmentation.

**Table 3:** Comparison of 3D U-Net segmentation model and *KneeXNet-2.5D* and *KneeXNet-2.5D-Baseline* in terms of IoU, DSC, training time, and inference time

Models	IoU	DSC	Training Time	Training Memory	Inference Time	Inference Memory
KneeXNet-2.5D-Baseline	0.8021	0.8721	81.32 min	1.34 GB	0.42 s	0.35 GB
KneeXNet-2.5D	0.8108	0.8779	569.30 min	10.42 GB	8.91 s	2.38 GB
3D U-Net	0.5428	0.5706	1357.49 min	6.53 GB	0.50 s	0.64 GB

Time is reported in minutes (min) for training and seconds (s) for inference. Inference time is measured per full knee MRI scan. Memory usage is measured in gigabytes (GB) based on peak GPU allocation.

### Comparison with other works in the literature

We benchmarked our proposed *KneeXNet-2.5D* against several state-of-the-art 2D, 2.5D, and 3D deep learning models for knee cartilage and meniscus segmentation. As summarized in Table 4, *KneeXNet-2.5D* achieves competitive segmentation accuracy with a DSC of 0.8779 and an IoU of 0.8108, outperforming other 2.5D and 3D approaches in overall cartilage segmentation. Compared to 3D models like *U-Net-CGAN*, which requires more memory and longer computation despite similar accuracy for certain meniscus regions, *KneeXNet-2.5D* provides an efficient trade-off between segmentation performance and computational cost. Moreover, unlike 2D models, which process slices independently and may lose inter-slice contextual information, *KneeXNet-2.5D* captures local 3D context, resulting in more precise segmentation boundaries and consistent predictions across slices. These characteristics make *KneeXNet-2.5D* particularly suitable for rapid and high-throughput knee MRI analysis, highlighting its potential for clinical research, deployment, and large-scale studies.

**Table 4:** Quantitative comparison of deep learning models for knee cartilage and meniscus segmentation, including *KneeXNet-2.5D* and selected methods from the literature

Model	Dataset	Method	IoU	DSC
**KneeXNet-2.5D**	218 patients	2.5D	0.8108	0.8779
SaMRI-2^74^	327 patients	3D	0.843	0.731
U-Net-CGAN^75^	88 patients	3D	–	0.895 (MM), 0.874 (LM)
3D U-Net^76^	88 patients	3D	–	0.831 (MM), 0.883 (LM)
2D U-Net^37^	638 patients	2D	–	0.662 (LM), 0.707 (MM) (T1*ρ*) 0.812 (LM), 0.731 (MM) (weDESS)

MM and LM refer to the *medial meniscus* and *lateral meniscus*, respectively. The terms T1*ρ* and weDESS indicate two MRI sequences: (1) Proton Density-Weighted Imaging, and (2) Weighted Echo Double-Echo Steady State Imaging. The dataset represents the number of patients included in each study. The terms 3D, 2D, and 2.5D specify the dimensionality of the method used for segmentation.

### *KneeXNet-2.5D*: app interface overview

**Fig. 7:**
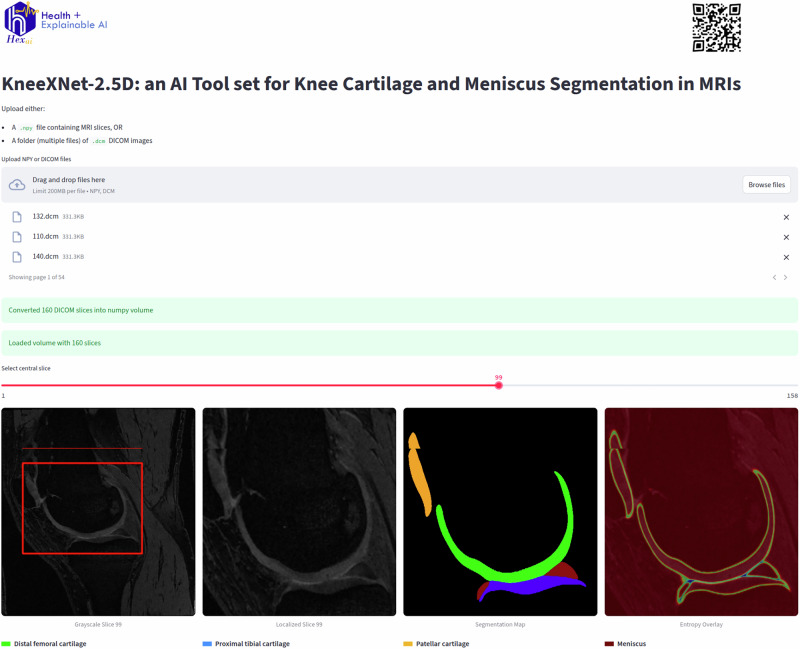
Application interface. The lightweight interface is built using Streamlit to facilitate real-time interaction and visualization of knee cartilage and meniscus segmentation results produced by *KneeXNet-2.5D*. Designed for accessibility, the interface operates entirely through a standard web browser without the need for high-performance computing resources, making it suitable for deployment in hospitals, academic settings, or rural health centers with limited computational infrastructure.

Figure 7 illustrates our lightweight and interactive software application designed to deploy *KneeXNet-2.5D* in both clinical and research environments. To bridge the gap between research results and real-world usability, this application supports real-time visualization of segmentation outputs, integrates entropy-based uncertainty maps, and enables streamlined interaction for domain experts, making it particularly suitable for routine use in musculoskeletal imaging workflows. The interface was built using Streamlit^36^, enabling accessibility through a simple web browser without requiring specialized software or high-end computational infrastructure. Users can upload knee sagittal MRI slices. Once loaded, the software app provides intuitive navigation through the MRI volume, allowing clinicians and researchers to scroll through slices and directly visualize model outputs.

The application integrates the complete *KneeXNet-2.5D* pipeline described in the study. First, a YOLO-based localizer automatically detects and crops the knee joint region of interest. Subsequently, multiple U-Net-based 2.5D segmentation models (trained at both 256 × 256 and 512 × 512 resolutions with Gaussian noise augmentations) are applied to generate probability maps. These predictions are fused to produce the final segmentation masks for the distal femoral cartilage, the proximal tibial cartilage, the patellar cartilage, and the meniscus. The results are color-coded and displayed alongside the original grayscale slice and localized knee joint area. A unique feature of the interface is its AI explainability module, which overlays entropy-based uncertainty maps directly onto the input images. This allows users to interpret the confidence of the predictions and identify regions where the model is uncertain, often corresponding to clinically ambiguous or anatomically complex areas. Together, these components offer a transparent workflow in which users can view the original input, localized ROI, predicted segmentation, and uncertainty map in parallel.

To further enhance usability, the app includes a built-in legend for segmentation classes, consistent visualization settings, and lightweight resource requirements, making it deployable even in low-resource healthcare environments. By combining robust back-end deep learning models with a simple, user-oriented interface, the KneeXNet-2.5D app represents a practical tool for supporting knee MRI analysis and facilitating adoption in clinical and research workflows.

## Discussion

This study introduced *KneeXNet-2.5D*, a clinically oriented and explainable deep learning framework for accurate segmentation of knee cartilage and meniscus in sagittal MRI scans. Our framework was designed to bridge the gap between high-performance AI models and their practical utility in orthopedic imaging workflows, by addressing three key pillars: segmentation accuracy, AI model robustness, and AI explainability.

Clinically, the proposed method holds promise for aiding follow-up and treatment monitoring in a range of orthopedic scenarios. This includes early detection and progression tracking of knee OA, evaluation of cartilage integrity after sports injuries in younger populations, and identification and progression of early and moderate OA in the elderly. By automating segmentation, our approach helps streamline radiological assessments and support timely interventions in both routine and longitudinal care. Moreover, the efficient design of the 2.5D segmentation model allows for reduced resource consumption, making it suitable for deployment in any healthcare settings with limited computational infrastructure. This is particularly relevant in low-resource hospitals and rural care environments, where timely access to specialist interpretation may be limited.

Implementing automated knee MRI segmentation systems, such as our proposed *KneeXNet-2.5D*, has substantial health system and economic implications. Manual cartilage segmentation traditionally requires 30–60 min per case, whereas radiologist interpretation of knee MRI typically takes 8–11 min. Automated systems reduce this process to seconds or a few minutes per scan^37–43^. This efficiency enables higher throughput, decreases radiologist fatigue, and allows reallocation of radiologist time toward more complex interpretive tasks^44^. Given the high annual volume of TKA procedures and the growing use of knee MRI for OA assessment, automation offers the scalability required for population-level quantitative imaging, supporting both clinical decision-making and research^45,46^. Although radiologist compensation and Medicare reimbursement for knee MRI, identified by the Current Procedural Terminology (CPT) code 73721, are fixed per study, reducing interpretation time enables practices to increase patient throughput without incurring additional staffing costs. Deep learning systems, such as *KneeXNet-2.5D*, have demonstrated segmentation speeds of just 5–90 s per scan, while maintaining accuracy comparable to manual methods, with cartilage DSC frequently exceeding 0.85.^37–43^. These gains translate into significant labor cost savings and facilitate the wider adoption of quantitative MRI biomarkers in monitoring knee OA progression and planning surgical interventions^45,47^. Knee OA itself is a major driver of healthcare expenditures in established economies. Direct medical costs per patient often exceed $7000–$15,000 annually, with additional indirect costs due to productivity loss and increased utilization of outpatient, pharmacy, and surgical services^48–50^. The American Society of Pain & Neuroscience^51^ reports that knee OA accounts for more than $12,000 per patient annually in direct and indirect costs, while total knee replacement procedures cost ~$16,000 per discharge, representing billions in cumulative inpatient expenditures^49^. Beyond cost, OA contributes to early workforce exit and substantial quality-adjusted life year (QALY) losses^49,50^.

Our model demonstrated strong performance in segmentation tasks (Table 1), achieving high mean IoU and DSC scores across key anatomical structures. These results were achieved using a 2.5D architecture that balances spatial contextual awareness with computational efficiency, offering a viable alternative to more resource-intensive 3D models. To enhance generalizability and model reliability in real-world clinical environments, we implemented a targeted image augmentation strategy that included synthetic noise injection using Gaussian blur (Fig. 1). The robustness evaluations confirmed the model’s stability under varying image conditions, a critical factor for MRI scans acquired using different machines or protocols. We also addressed the growing demand for transparency in AI applications within healthcare by incorporating an entropy-based explainability method. The generated uncertainty maps not only provided visual insights into prediction confidence, but were also validated through domain-expert-in-the-loop evaluations. In addition to computational analysis of AI interpretability through faithfulness (Table 2), we engaged two board-certified orthopedic surgeons to independently validate our AI explainability model. Their expert evaluation confirmed that regions with high entropy (Fig. 6), indicative of model uncertainty, often aligned with anatomically or clinically ambiguous areas. This alignment supports both the faithfulness and interpretability of our AI model, reinforcing that its decision-making process reflects real-world clinical reasoning.

In this study, we intentionally selected a U-Net with a ResNet-34 encoder to align with our clinical and translational goals. Our dataset size is not well suited for training transformer-based or attention-heavy deep learning architectures, which generally require much larger imaging cohorts to mitigate overfitting. Moreover, a core aim of our proposed KneeXNet-2.5D is deployability in resource-limited clinical settings, where transformer models, with their higher memory demands, longer inference times, and larger parameter counts, are less practical. The ResNet-34 U-Net backbone provides a stable, efficient, and high-performing foundation, allowing us to isolate and showcase the contributions of our 2.5D *ensemble design* and *scale-space representation* strategies without introducing unnecessary architectural complexity.

To encourage adoption and reproducibility, we publicly released the entire pipeline, including a manually annotated gold-standard knee MRI segmentation dataset, annotation guidelines, model weights, source code, and a lightweight software application. This tool streamlines real-world deployment and integration into existing musculoskeletal imaging workflows, lowering barriers to clinical use and academic experimentation.

Despite the promising results, a few limitations should be acknowledged. Our AI models were trained and evaluated on sagittal MRI scans only, and from a single source imaging repository, namely the Osteoarthritis Initiative (OAI)^52^. Therefore, external validation on diverse datasets remains a necessary step. Future work will focus on evaluating *KneeXNet-2.5D* on harmonized public datasets and incorporating more advanced architectures once a sufficient number of annotated volumes become available. Additionally, while the entropy-based explainability strategy offered quantitative insight, integrating complementary explainability methods (e.g., saliency maps or attention mechanisms) could further strengthen and generalize the model interpretability. Finally, although we included expert evaluation, future studies should consider user-centered clinical trials to better assess how such tools influence diagnostic confidence and decision-making. Future work will focus on expanding the model to handle multi-view MRI inputs and integrating it into Picture Archiving and Communication System systems for real-time segmentation. From a clinical perspective, studies involving radiologists and orthopedic surgeons in practical deployment scenarios will be critical to evaluate its utility, usability, and long-term adoption. Additionally, we aim to extend the framework into a unified segmentation system capable of handling both knee and hip joint structures, supporting broader musculoskeletal applications. This universal segmentation approach would enable shared model architecture and training pipelines across joint types, further enhancing scalability and reducing development redundancy.

## Methods

In this study, we introduce *KneeXNet-2.5D*, an explainable deep learning framework for accurate and computationally efficient segmentation of knee cartilage and meniscus in sagittal MRI scans. The proposed approach combines the spatial contextual awareness of 3D models with the efficiency and scalability of 2D architectures through a 2.5D design, while also incorporating model robustness and AI explainability. The complete pipeline, including dataset, data pre-processing, knee joint area localization, segmentation, and AI explainability, is illustrated in Fig. 1.

### Dataset and manual annotation

The dataset was obtained from the OAI^52,53^, a publicly available and longitudinal cohort that provides high-quality knee radiographs and MRIs, combined with clinical and demographic data. Our study did not require Institutional Review Board (IRB) approval, as it involved only secondary analysis of de-identified and publicly available data from the OAI^52,53^. This study did not involve the recruitment of new participants or any direct interaction with human subjects. Instead, it utilized de-identified and publicly available data from the OAI^52,53^, a large multi-center and longitudinal study. Ethical oversight for participant consent and data sharing was handled by the OAI coordinating centers in accordance with institutional guidelines. As such, no additional informed consent was required for our secondary analysis. As confirmed by the University of Pittsburgh Human Research Protection Office, research using only publicly available data is not considered human subjects research and does not require IRB review or approval.

**Fig. 8:**
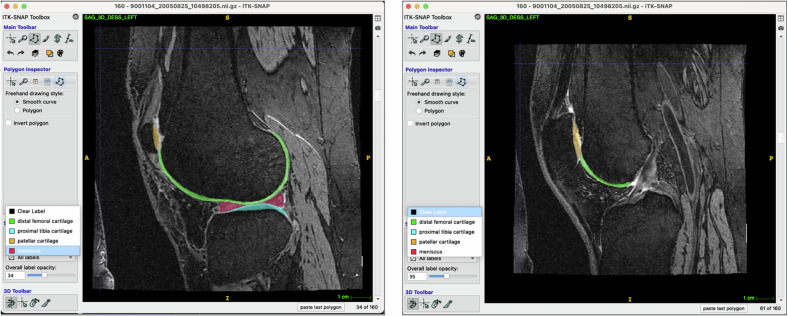
A representative MRI slice manually annotated using ITK-SNAP^54^ (two representative examples), illustrating the distal femoral cartilage, proximal tibial cartilage, patellar cartilage, and meniscus.

In this study, the MRI scan parameters were: field of view = 14.0 cm × 14.0 cm, matrix size = 384 × 384, echo time = 4.71 ms, repetition time = 16.32 ms, with 160 slices at a thickness of 0.7 mm. For this study, we selected a subset of 218 participants, corresponding to 222 MRI scans, based on the quality of their baseline T2-weighted sagittal knee MRIs. This set included 4 participants with imaging of both the left and right knees. The T2-weighted sequences provide clear delineation of cartilage, meniscus, and surrounding soft tissues, enabling detailed morphological assessment. T2-weighted sequences were specifically chosen for their strong contrast between cartilage, bone, and synovial fluid, which facilitates accurate manual segmentation of knee joint structures. Among 218 unique patients, 68.8% identified as White or Caucasian, 28.4% as Black or African American, 2.3% as Other Non-white, and 0.5% as Asian. The sex distribution included 57.8% female patients and 42.2% male patients. In our study, 50% of patients were aged 41–60 years, 29.36% were 61–70 years, and 20.64% were older than 70 years. Each participant’s unique identifier was retained to allow potential longitudinal tracking. Manual segmentation was performed on each MRI slice to label four key anatomical structures, including: (1) distal femoral cartilage, (2) proximal tibial cartilage, (3) patellar cartilage, and (4) the meniscus. All annotations were conducted using ITK-SNAP^54,55^, an open-source and interactive tool for medical image segmentation. The annotation process was carried out using an annotation guideline developed by a domain expert. All processing was done in grayscale mode to enhance boundary visibility and improve labeling precision. Five trained annotators were involved in the labeling process, each with prior experience in musculoskeletal image annotation. The annotators generally began segmentation from the point where the relevant joint structures became clearly visible, typically after scrolling past the surrounding soft tissues, such as muscle. The first identifiable structures were often the meniscus and femoral cartilage, followed by the tibial cartilage and patellar cartilage. Annotators were instructed to segment all slices in which any of these structures appeared. To assess inter-rater agreement, a batch of 10 knee MRIs was independently segmented by all five annotators. The average pairwise IoU^26^ and DSC^27^ across the four anatomical structures was calculated, yielding a mean IoU and DSC of 0.79 and 0.85, respectively, indicating high consistency and reproducibility among annotators. All segmentation was reviewed under the supervision of two orthopedic surgeons to ensure anatomical validity. This served as a gold standard for training and evaluating the deep learning models. An example of the manual annotation is shown in Fig. 8.

### Data pre-processing

To localize the knee joint area, we implemented a two-stage pre-processing pipeline. First, following a predefined guideline to ensure consistency, a trained annotator manually applied bounding box annotations to a representative subset of sagittal knee MRI scans using Label Studio^56^. These annotations were then used to train a YOLOv11-based object detection model^24,25^, which automatically generated bounding boxes across the remaining dataset. The predicted bounding boxes were used to crop the MRI images, focusing exclusively on the relevant joint regions (see Fig. 1). This step effectively removed irrelevant anatomical areas and emphasized local structural features, allowing the segmentation model to concentrate on the cartilage and meniscus while reducing background noise and computational complexity.

### Deep learning architecture

The segmentation model was built using a 2.5D U-Net architecture with a ResNet-34 encoder^20,57^. The encoder enhanced feature extraction through deep residual connections while maintaining efficiency and reducing dependency on large-scale training data. The model accepted three input channels, which were constructed by stacking a target sagittal MRI slice with its immediate anterior and posterior neighbors (slices *z* − 1, *z*, and *z* + 1). This configuration captures local spatial context across adjacent slices while avoiding the computational overhead of full 3D convolutions. The current deep learning architecture produced a five-class segmentation map corresponding to four anatomical structures, including distal femoral cartilage, proximal tibial cartilage, patellar cartilage, and meniscus, plus one background class.

### Model training and image augmentation

The dataset was randomly split into training (80%), validation (10%), and testing (10%) subsets, comprising 19,702 slices for training, 2463 slices for validation, and 2463 slices for testing. The model was trained for 30 epochs using the Adam optimizer with a learning rate of 1 × 10^−4^, and this learning rate was not tuned. The model was optimized using a standard cross-entropy loss. The batch size was fixed at 8 for all experiments, and no search over alternative batch sizes was performed. The number of epochs was also kept fixed rather than selected through a tuning process. No weight decay or other forms of explicit regularization were applied. Data samples were shuffled at each epoch, but all other hyperparameters remained constant across runs. For *KneeXNet-2.5D-Baseline*, slices and masks were symmetrically zero-padded to the largest in-stack dimensions. Batches were then zero-padded to the next multiple of 32 in height and width to match encoder downsampling; images were not resized. To improve model robustness and adaptability to variability in medical imaging, we introduce *KneeXNet-2.5D* and applied data augmentation during both the training and validation phases, using controlled Gaussian blur^58,59^ as our primary augmentation technique. Clinical MRI slices often exhibit heterogeneity in noise levels and smoothness across scanners and imaging sessions. Applying the same mild, deterministic blur to both training and validation samples stabilizes the preprocessing pipeline and ensures that evaluation is performed under the same noise conditions modeled during training. This approach supports more consistent estimates of model stability without altering anatomical content. Importantly, no augmentation was applied to the test set, ensuring that all final performance metrics reflect performance on unmodified images. Four independent models were parallel trained, each using a distinct Gaussian blur configuration characterized by a combination of input resolution (256 × 256 or 512 × 512), kernel size (*k* = 5 or 7), and standard deviation (*σ* = 0.10 or 0.15) (see Fig. 1). These combinations were chosen to simulate varying degrees of image smoothness and spatial degradation, capturing the variability commonly observed in different clinical imaging conditions.

The trained AI models from the *KneeXNet-2.5D* ensemble, intended to achieve robust segmentation of knee joint tissues. Softmax probability maps were generated for each image by passing them through four separately trained models. Probability maps from the models trained on 256 × 256 inputs are resized to 512 × 512 to ensure spatial alignment with the native 512 × 512 outputs. We then perform an element-wise average of the aligned probability maps from all models to obtain a fused probability map, a commonly used ensemble strategy in medical image segmentation^60,61^. The final segmentation is derived by assigning to each pixel the class corresponding to the highest probability in the fused map. By integrating outputs from diverse augmentation conditions, this aggregation strategy reduces sensitivity to input perturbations and leads to more stable and reliable segmentation predictions.

### Timing and memory measurements

We measured runtime and GPU memory to enable transparent and reproducible comparisons. Training time is defined as the wall-clock duration per model from the first to the last parameter update. *KneeXNet-2.5D* consists of four independent models (distinct data-augmentation configurations), trained in parallel on separate GPUs. Training memory usage is the peak GPU allocation recorded during training. Inference time was measured per full knee MRI volume (160 slices), defined as the duration to process the scan and produce the segmentation mask. Inference memory usage denotes the peak GPU memory consumed during this process.

### AI explainability and interpretability

There is a pressing need to develop AI methods that are both explainable and interpretable, particularly in healthcare and orthopedic applications^62–65^. To incorporate deep learning explainability of the segmentation model, we utilized an entropy-based approach that quantifies pixel-wise uncertainty^33–35,62^. For each test sample, we computed an entropy map using the fused softmax probability map, which was obtained by averaging the outputs of four independently trained models. The Shannon entropy^33^ at each pixel was calculated as Eq. (1):

1 $${\rm{Entropy}}\,(x,y)=-\mathop{\sum }\limits_{c=1}^{C}{p}_{c}(x,y)\cdot \log ({p}_{c}(x,y)+\varepsilon )$$

where *p*_*c*_(*x*, *y*) denotes the softmax probability of class *c* at spatial location (*x*, *y*). *C* is the number of segmentation classes, and *ε* = 10^−8^ is added for numerical stability. To facilitate interpretation, the entropy map was visualized as an overlay on the corresponding input image. Entropy values were masked to include only foreground regions (e.g., where the ground truth label was non-zero), ensuring the visualization focused on anatomically relevant structures. The masked entropy values were linearly normalized for display, and both the visual overlays and raw entropy arrays were saved for further analysis. As shown in Fig. 6, this method provides a spatial representation of model uncertainty and supports the identification of regions where predictions are less confident, enabling a qualitative assessment of the model’s decision-making behavior^34,62^.

### Evaluation strategies

This section outlines the evaluation strategies employed to comprehensively assess the proposed framework across three critical dimensions, including: (1) segmentation accuracy, (2) model robustness, and (3) model explainability. Accurate segmentation is essential for reliable analysis and interpretation in medical imaging tasks; however, performance on clean and ideal data alone is insufficient to gauge a model’s reliability in real-world clinical settings. Model robustness evaluation is therefore equally important, as MRI inputs are often subject to variability and noise introduced during acquisition. By jointly evaluating segmentation performance and robustness under both standard and perturbed conditions, we aim to establish a more complete understanding of the model’s utility in practical deployments. Additionally, we incorporate AI explainability assessments to ensure that the model’s predictions are based on anatomically meaningful features, thereby enhancing model interpretability and clinical trust.

**Localization and segmentation evaluation:** To quantify the spatial overlap between predicted localization and/or segmentation masks and ground truth annotations, we employed two widely recognized metrics: IoU^26^ and DSC^27^, both standard in medical image analysis^66–68^. These metrics are basically defined in Eq. (2) and Eq. (3) as follows:

2 $$\,{\rm{IoU}}=\frac{| {\rm{Prediction}}\cap {\rm{Ground\; Truth}}| }{| {\rm{Prediction}}\cup {\rm{Ground\; Truth}}\,| }$$

3 $$\,{\rm{DSC}}=\frac{2\times | {\rm{Prediction}}\cap {\rm{Ground\; Truth}}| }{| {\rm{Prediction}}| +| {\rm{Ground\; Truth}}\,| }$$

Both metrics range from 0 to 1, with higher values indicating better spatial agreement. IoU tends to penalize over-segmentation more strongly than DSC, making it particularly sensitive to false positives. When used together, IoU and DSC offer complementary insights into segmentation performance and provide a robust basis for evaluating model accuracy.

**Robustness evaluation:** We utilized a set of metrics to quantify model robustness under image augmentation and its capacity to recover performance using augmentation-based methods. The evaluation framework is well-structured to systematically assess model robustness and recovery performance under various augmentation scenarios.

- **Relative performance drop (RPD)**. The RPD quantifies the proportional reduction in segmentation when augmentation is applied, relative to baseline performance in clean data. Similar performance degradation metrics have been used in robustness studies for vision models^29,30^. As defined in Eq. (4) and Eq. (5), it is computed separately for the the IoU and Dice metrics for each augmentation condition *i*.
  4 $${\,{\rm{RPD}}}_{{\rm{IoU}}}^{(i)}=\frac{{{\rm{IoU}}}_{{\rm{clean}}}-{{\rm{IoU}}}_{{\rm{aug}}}^{(i)}}{{{\rm{IoU}}}_{{\rm{clean}}}}$$
  5 $${\,{\rm{RPD}}}_{{\rm{Dice}}}^{(i)}=\frac{{{\rm{Dice}}}_{{\rm{clean}}}-{{\rm{Dice}}}_{{\rm{aug}}}^{(i)}}{{{\rm{Dice}}}_{{\rm{clean}}}}$$
- **Robustness Index (RI)**. The RI, defined in Eq. (6), quantifies the model’s ability to maintain performance under augmentation mechanisms. Similar metrics for evaluating robustness under input corruptions and distribution shifts have been proposed in prior studies^30,31^. It is computed separately for each augmentation condition *i*.
  6 $${{\rm{RI}}}^{(i)}=0.5\cdot \left(1-{\,{\rm{RPD}}}_{{\rm{IoU}}}^{(i)}\right)+0.5\cdot \left(1-{\,{\rm{RPD}}}_{{\rm{Dice}}}^{(i)}\right)$$
- **Performance Recovery Ratio (PRR)**. The PRR quantifies the degree to which the model regains its original segmentation performance on clean data following augmentation-based recovery^32^, as given in Eq. (7) and Eq. (8):
  7 $${{\rm{PRR}}}_{{\rm{IoU}}}=\frac{{{\rm{IoU}}}_{{\rm{recovered}}}}{{{\rm{IoU}}}_{{\rm{clean}}}}$$
  8 $${{\rm{PRR}}}_{{\rm{Dice}}}=\frac{{{\rm{Dice}}}_{{\rm{recovered}}}}{{{\rm{Dice}}}_{{\rm{clean}}}}$$
- **Composite Robustness-Recovery Score (CRRS)**. The CRRS integrates robustness and recovery performance into a single measure by incorporating the average RPD across all augmentation conditions, as shown in Eq. (9) and Eq. (10):
  9 $${\overline{{\rm{RPD}}}}_{{\rm{IoU}}}=\frac{1}{N}\mathop{\sum }\limits_{i=1}^{N}{{\rm{RPD}}}_{{\rm{IoU}}}^{(i)}$$
  10 $${\overline{{\rm{RPD}}}}_{{\rm{Dice}}}=\frac{1}{N}\mathop{\sum }\limits_{i=1}^{N}{{\rm{RPD}}}_{{\rm{Dice}}}^{(i)}$$
  The CRRS is then computed by combining recovery and robustness terms, as shown in Eq. (11):
  11 $$\,{\rm{CRRS}}=\lambda \cdot {{\rm{PRR}}}_{{\rm{IoU}}}+(1-\lambda )\cdot {{\rm{PRR}}}_{{\rm{Dice}}}-0.5\cdot \left({\overline{{\rm{RPD}}}}_{{\rm{IoU}}}+{\overline{{\rm{RPD}}}}_{{\rm{Dice}}}\right)$$
  Here, *λ* ∈ [0, 1] is a weighting parameter that balances the contributions of IoU and Dice recovery in the final score. In our study, we set *λ* = 0.5 to give equal importance to both terms.

**AI Explainability Evaluation:** To evaluate and measure AI explainability, we employed two complementary strategies: (1) entropy-based faithfulness analysis^69–71^, and (2) domain-experts-in-the-loop assessment.

- **Entropy-Based Faithfulness Analysis:**We implemented an entropy-guided approach to quantify the faithfulness of the model’s predictions. Specifically, for each test image, we computed a fused softmax probability map by averaging outputs from four independently trained models. From this, a pixel-wise entropy map was derived to highlight regions of predictive uncertainty^34^. The top 20% of high-entropy pixels were identified as the most informative and potentially decision-critical areas^72^. To assess the model’s reliance on these regions, we selectively corrupted them using Gaussian noise and reprocessed the perturbed images through the ensemble. The resulting segmentations were then compared to the original outputs using IoU and DSC. A measurable performance drop under targeted corruption was interpreted as evidence that the model was relying on clinically meaningful, high-uncertainty regions, thereby supporting the faithfulness of its predictions^62,73^ (see Table 2).
- **Domain-Experts-in-the-Loop Assessment:** To further validate the interpretability and clinical relevance of the model’s predictions, we engaged two board-certified orthopedic surgeons specializing in knee disorders. Each expert independently reviewed a random subset of 15 test MRI scans alongside the corresponding segmentation outputs and associated entropy maps.

## Data Availability

Data, image annotation guideline(s), and the annotated MRI imaging dataset supporting the findings of this study are publicly and freely available at https://github.com/pitthexai/Knee_MRI_Segmentation_2.5D. The original knee MRI scans can be accessed through the Osteoarthritis Initiative (OAI) dataset portal at https://nda.nih.gov/oai^52^.

## Code availability

The source code, model weights, and a lightweight software application are publicly and freely available for any research, educational, and academic purposes at: https://github.com/pitthexai/Knee_MRI_Segmentation_2.5D.
